# Retinoids in Cutaneous Squamous Cell Carcinoma

**DOI:** 10.3390/nu13010153

**Published:** 2021-01-05

**Authors:** Helen B. Everts, Eleonore-Nausica Akuailou

**Affiliations:** Department of Nutrition and Food Sciences, Texas Woman’s University, Denton, TX 76209, USA; eakuailou@twu.edu

**Keywords:** vitamin A, skin cancer, retinoids, metabolism, retinoid resistance

## Abstract

Animal studies as early as the 1920s suggested that vitamin A deficiency leads to squamous cell metaplasia in numerous epithelial tissues including the skin. However, humans usually die from vitamin A deficiency before cancers have time to develop. A recent long-term cohort study found that high dietary vitamin A reduced the risk of cutaneous squamous cell carcinoma (cSCC). cSCC is a form of nonmelanoma skin cancer that primarily occurs from excess exposure to ultraviolet light B (UVB). These cancers are expensive to treat and can lead to metastasis and death. Oral synthetic retinoids prevent the reoccurrence of cSCC, but side effects limit their use in chemoprevention. Several proteins involved in vitamin A metabolism and signaling are altered in cSCC, which may lead to retinoid resistance. The expression of vitamin A metabolism proteins may also have prognostic value. This article reviews what is known about natural and synthetic retinoids and their metabolism in cSCC.

## 1. Introduction

Cutaneous squamous cell carcinoma (cSCC) is a form of keratinocyte carcinoma, also known as non-melanoma skin cancer. The primary extrinsic etiological factor for the development of cSCC is the chronic lifetime exposure to solar ultraviolet light radiation (UVR) and to indoor artificial UV light-induced tanning [1,2]. Among the solar UVR components, UVB (290–320 nm) is considered mostly responsible for UV-induced carcinogenesis [1]. However, cSCC can also form from papilloma virus (PV) infection [2,3]. Precancerous lesions include warts, actinic keratosis (AK), keratoacanthomas (KA), and porokeratosis [4]. While cSCC is seen in the upper cells of the epidermis, this cancer originates from aberrant regulation of hair follicle stem cells [5,6,7,8]. Keratinocyte carcinomas are the most common form of cancer in humans, with over 1 million Medicare patients treated annually in the U.S. [9]. A population-based study conducted in Minnesota revealed a rise of 263% in cSCC incidence between 1976–1984 and 2000–2010 [10]. Americans spent $4.8 billion per year on nonmelanoma skin cancer treatment between 2007 and 2011 and $1.68 billion per year (2013) on treating the precursor lesion AK [11,12]. Standard treatment of cSCC is surgical removal and/or radiation, which is effective in most patients. The “cure” is temporary: 91% of patients who have cSCCs surgically removed develop an additional tumor within 10 years, and high-risk patients have greater tumor recurrence [13]. Metastasis of cSCC occurs in 2–5% of all cSCC patients and 4–16% of high-risk patients: the mortality rate of metastasized cSCC is over 70% [14,15,16]. In the southern half of the U.S., death from cSCC is similar to several other cancers and higher than melanoma, renal, and oropharyngeal carcinomas [15]. This high risk is due to the presence of an aggressive tumor, multiple tumors, or immunosuppressed patients [17]. In immunosuppressed organ transplant recipients, the risk of cSCC is 65 times greater than the general population. Early detection of high-risk aggressive tumors leads to better treatments.

Retinoids are a family of natural and synthetic vitamin A derived compounds. Natural forms include retinyl esters, retinol, retinal, and retinoid acid (RA; Table 1). Retinyl esters are the main storage form and dietary source from animals [18]. Additional dietary sources include the provitamin A carotenoids: beta-carotene, alpha-carotene, and beta-cryptoxanthin. Retinol, bound to retinol binding protein 4 (RBP4), is the predominate circulating form of vitamin A [19] and its circulating concentrations are tightly controlled [20]. 11-*cis* retinal, 9-*cis* RA, and all-*trans* RA are the active forms of vitamin A. Skin also contains a unique retinoid: 3,4-didehydroretinol (ddRetinol), with its corresponding ester (ddretinyl ester), aldehyde (ddretinal), and carboxylic acid (ddRA) [21,22,23]. Several synthetic retinoids have also been developed and are used in the treatment of many dermatological diseases including cSCC (Table 2) [4,24]. The purpose of this article is to review the interactions between these retinoids and cSCC.

## 2. Vitamin A Metabolism and Signaling

RA synthesis occurs in, or near, the cells in which it will ultimately be used. Precise spatial and temporal control of RA levels in skin is achieved by regulating a few key steps in cellular vitamin A metabolism. Retinol enters the cell by passive diffusion or the transport proteins known as stimulated by RA6 (STRA6) [40] and RBP receptor 2 (RBPR2) [41]. In the skin, most retinol that enters the keratinocyte is stored as retinyl esters by the action of lectin:retinol acyltransferase (LRAT) [42,43] or acyl-CoA:diacylglycerol acyltransferase 1 (DGAT1) [44]. The remaining retinol is reversibly oxidized into retinal by retinol dehydrogenases of the short chain dehydrogenase/reductase (SDR) superfamily [27,45]. Five members of this family that localize to the skin include dehydrogenase reductase SDR family member 9 (DHRS9) [46,47,48,49], retinol dehydrogenases 1/16 (RDH1/16) [34,50], RDHE2, RDHE2-similar [51,52], and RDH10 [35,53]. Cellular retinol binding proteins (RBP 1-2) deliver retinol to LRAT and these SDRs [26]. DHRS3 is also in this SDR superfamily, but catalyzes the reverse reaction to prevent RA toxicity [54]. Retinal is subsequently oxidized to RA by retinal dehydrogenases 1–3 (ALDH1A1, 2, and 3) [26]. Cellular RA binding protein 1 (CRABP1) guides RA to its catabolism process, which is achieved by the action of cytochrome P450 26 family members (CYP26A1, B1, and C1) [26,55,56,57,58]. However, the function of RA metabolites is still a subject of controversy [59,60,61]. These CYP26 enzymes maintain RA levels through an inhibitory feedback loop [62]. High levels of RA directly induce CYP26A1 and CYP26B1 in the skin [63,64,65], which then degrades this excess RA. During development, CYP26 family members create boundaries to reduce the spread of RA [66]. On the other hand, cellular RA binding protein 2 (CRABP2) protects RA from degradation by CYP26 enzymes, while it chaperones RA to the nucleus and channels it to retinoic acid receptors alpha (RARA) to enhance transcriptional activity [67,68,69]. It is not known if CRABP2 may also bind RARB and RARG, as only RARA was tested. A spatial and temporal correlation was also established between CRABP2 expression and RA synthesis [70,71,72]. The actions of RA binding proteins may not be as clear-cut, as CRABP2 also binds CYP26B1 to facilitate RA catabolism in vitro [58]; and CRABP1 carries RA into the nucleus and releases RA for binding to RARs without channeling it directly [67]. These additional functions may be necessary because these proteins do not always localize to the same cells [26,73]. Since retinoids are hydrophobic, retinoid metabolons form to complex the enzymes and binding proteins [26]. Each metabolon has a complete set of enzymes and binding proteins, but different family members of these proteins can form different metabolons to allow for differential regulation. All components necessary for RA synthesis, degradation, and signaling localize to the skin [47,73,74,75,76].

ddRetinol is irreversibly synthesized from retinol in keratinocytes [77,78,79]. Cytochrome P450 family member 27C1 (CYP27C1) is the sole enzyme required for ddretinoid synthesis in zebrafish [29]. CYP27C1 localizes to human skin mitochondria and converts retinol to ddretinol in vitro [22,23]. However, *Cyp27c1* is not present in the mouse genome [80] even though ddretinoids increased in skin tumors from UVB-induced cSCC in hairless mice [81]. Like retinol, ddretinol binds CRBP1 and RBP4 [32], is esterified by LRAT [33], and is oxidized into retinal by RDH1/16 [34], and possibly RDH10 [35]. In addition, ddRA binds CRABP2 [36]. It is unclear if DHRS9 converts ddretinol to ddretinal, and whether ALDH1A1, ALDH1A2, or ALDH1A3 converts ddretinal to ddRA.

RARs are RA dependent transcription factors of the nuclear hormone family [82,83] that regulate the expression of >500 genes involved in differentiation, cell cycle control, and apoptosis either directly or indirectly [84]. RA also regulates its own metabolism by regulating STRA6, RBP1, LRAT, DHRS9, DHRS3, RDH1/16, ALDH1A3, and CRABP2 as well as CYP26A1 and CYP26B1 discussed above [50,64,65,85,86,87,88,89,90,91]. Note that RA signaling is not this simple as RA can activate four additional signaling cascades. First, RA binding to membrane associated RARs leads to the phosphorylation of p38 MAPK [92]. This leads to the phosphorylation of MSK1, which goes on to phosphorylate and activate RARs in the nucleus. MSK1 and MAPKs also phosphorylate histones on target genes, corepressors, and coactivators. The overall effect is to increase the transcriptional activity of RA/RAR target genes. However, this signaling cascade also phosphorylates additional transcription factors leading to the activation of additional genes. Second, CRABP1 with RA directly binds RAF (rapidly accelerated fibrosarcoma) to modestly activate ERKs (extracellular signal-related kinases) [93]. RAF is a key component of epidermal growth factor (EGF) and other growth factor signaling. The EGF receptor is a tyrosine kinase, which activates rat sarcoma virus oncogene (RAS), which then binds and activates RAF. CRABP1 with RA also inhibited the binding of RAS to RAF, which led to a reduction of EGF and mutant overactive RAS induced ERK activation. Third, the entrance of retinol into the cell through STRA6 also triggers a signaling cascade by phosphorylating JAK2 [94]. Phosphorylated JAK2 goes onto phosphorylate STAT3 or STAT5 depending on the cell, which activates additional sets of genes. Fourth, when RA exceeds the capacity to bind CRABP2, it can bind fatty acid binding protein 5 (FABP5), which directs RA to peroxisome proliferator-activated receptor beta/delta (PPARB/D) and increases genes involved in proliferation [95,96]. However, other groups refuted this role of RA [97,98]. These multiple signaling pathways lead to diverse and sometimes confusing effects of RA, especially when given at pharmacological doses. 

The role of ddretinoids in mammalian skin is unclear. ddRA binds RARs at an affinity similar to RA [99]. Physiological levels of all-*trans* ddRA (10^−7^–10^−9^ M) activate RARB-RXRA heterodimers and RXRA-RXRA homodimers to a greater extent than all-*trans* RA [100]. Note that these reporter assays used the RARE from RARB, but work from Loraine Gudas’ laboratory shows that receptors work differently on different genes [101,102]. More recent studies in cultured keratinocytes reveal that pharmacological levels of ddRA and RA (10^−3^ M) regulated the same genes [88] while physiological concentrations (10^−9^ M) of ddRA and RA regulate different genes [103]. These studies suggest that ddRA is synthesized in skin and may alter a different set of genes based on the dose and receptor used. It is unclear if ddRA also binds PPARB/D in skin. It is also unknown which genes ddRA activates in vivo, as most of this work was done in monolayers of cultured cells and RA has different effects in vivo than in vitro [104]. 

## 3. Retinoids and Cutaneous Squamous Cell Carcinoma (cSCC)

**Figure 1:**
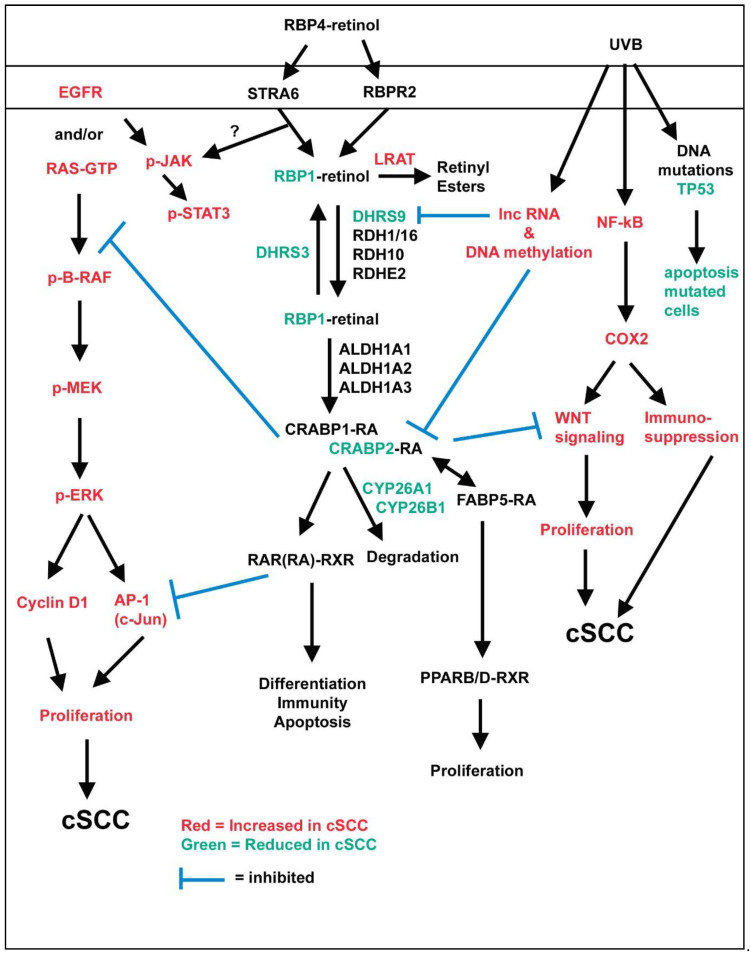
Interactions between retinoid metabolism and cutaneous squamous cell carcinoma (cSCC). This figure shows how both exogenous retinoic acid (RA) regulates epidermal growth factor (EGF) and Wingless-type MMTV integration site (WNT) signaling, and cSCC reduces the expression of key retinoid metabolism proteins. Items in red are increased in cSCC, items in green are decreased in cSCC. Arrows indicate signaling pathways. The blue blocked line indicates inhibition. ? indicates that it is unclear how STRA6 impacts STAT3 in the context of cSCC.

Vitamin A deficiency leads to squamous cell metaplasia in numerous epithelial tissues including the skin, hair, and sebaceous gland [105,106]. Topical RA inhibited papilloma formation in the chemical carcinogenesis mouse model where 7,12-dimethylbenz(*a*)anthracene (DMBA) initiates and 12-*O*-tetradecanoylphorbol-13-acetate (TPA) promotes tumorigenesis (Table 3) [107]. RA must be provided with or exactly 1 h prior to TPA to be effective. Topical RA inhibited ornithine decarboxylase activity [107], AP-1 activity [108], and EGF signaling (B-RAF/MEK/ERK MAP kinase pathway) upstream of STAT3 (Figure 1) [109,110]. However, microarray analysis also identified 31 genes related to cytokines and 12 genes related to WNT (wingless-type MMTV integration site) signaling that were regulated by RA, but were not studied further [109]. The inhibition of EGF signaling may be occurring via CRABP/RA binding to B-RAF, as CRABP1 binds B-RAF and inhibits EGF signaling [93]. In addition, RA treatment in DMBA/TPA exposed female *Crabp2^tm1Ipc^* null mice failed to reduce tumors [111]. These *Crabp2^tm1Ipc^* null mice also have a larger number and size of tumors, increased proliferation (Krt8 and Ki67), and reduced differentiation (Krt1/10). In addition, transfection of CRABP2 into HaCaT, FaDu, and A431 cells reduced EGF signaling. De Luca and colleagues showed that dietary RA inhibited both the promotion and progression of high-risk tumors promoted by mezerein [112], the progression of TPA promoted tumors [113], but had no effect when only DMBA was used [114]. The mechanism for this effect has not been determined. DMBA treated female and male mice that overexpressed CYP26A1 developed papillomas early and spontaneously developed invasive cSCC [115]. Retinoid metabolism proteins also increased in a DMBA-induced model of KA regression before WNT signaling was reduced [116]. High pharmacological levels (10uM) of RA increased two WNT inhibitors to reduce WNT signaling. This RA treatment resulted in regression of both KA and cSCC. These studies suggest that in DMBA/TPA-induced cSCC, exogenous RA inhibits tumor promotion by blocking overactive EGFR/RAS signaling, WNT signaling, and possibly other mechanisms. In addition, low endogenous RA levels lead to tumor promotion in the absence of a chemical tumor promoter, reduced differentiation, and increased proliferation. This suggests that maintaining endogenous RA levels is critical to the prevention of cSCC.

DMBA/TPA treatment leads primarily to RAS mutations, which are much less common in human cSCC [117]. The vast majority of cases of human cSCC are caused by chronic UVB exposure and TP53 mutations, which can be recapitulated in mice [117,118]. Results from the photocarcinogenesis model of cSCC using UVB or UVA&B treated hairless mice are even more confusing. Topical RA accelerated [119], inhibited [120], or had no effect [121] on photocarcinogenesis. These differences could be due to timing or dose of RA. These variable effects of RA may also be due to differences in the background strain of the hairless gene mutation, as different mouse strains have different susceptibility to cSCC and different levels of endogenous retinoids [122,123]. Oral retinol or the second-generation aromatic retinoid etretinate at two high doses also did not alter photocarcinogenesis in hairless mice [124]. These studies suggest a complex relationship between UVB, cSCC development, and RA signaling. In addition, chronic UV exposed hairless mice may be resistant to retinoids. This resistance limits studies needed to identify the mechanisms for how RA impacts UVB-induced cSCC. UVB exposure leads to TP53 mutations, reduced NOTCH signaling, excess COX2 activity, increased WNT signaling, and immunosuppression (Figure 1) [118,125,126,127,128,129]. RA regulates most of these pathways in other cells [130,131,132,133,134,135,136,137]. Therefore, RA may also regulate some of these pathways in UVB-induced cSCC.

In humans, oral acitretin (Soriatane, a derivative of etretinate) prevented cSCC reoccurrence in organ transplant patients, who have a high risk of cSCC reoccurrence [138]. In non-transplant patients, oral acitretin significantly reduced the number of tumors, but not the incidence or time to development of cSCC [139]. The authors argue this may be due to a small sample size. Acitretin is also used to treat AKs to prevent them from developing into cSCC [24,140]. Oral isotretinoin (Accutane; 13-*cis* RA) prevented reoccurrence of cSCC in xeroderma pigmentosum patients and BCC in patients with nevoid basal cell carcinoma (BCC syndrome) [39]. However, oral isotretinoin with interferon alpha was not effective at preventing the reoccurrence of cSCC in patients with aggressive disease [141]. Both of these oral retinoids have significant side effects that include mucocutaneous defects, skeletal hyperostosis, altered lipid profiles, hepatotoxicity, numerous ocular defects, and teratogenesis [39,142]. Furthermore, acitretin has the potential to be esterified to etretinate in the presence of alcohol [24]. Etretinate has the longest half-life at 80–160 days and can stay in the skin up to two years [143]. In contrast, the half-life of acitretin is 50 h, isotretinoin is 10–20 h, bexarotene is 7–9 h, and tretinoin is 40–60 min. Thus, the conversion of acitretin to etretinate significantly increases the amount of time the drug is in the body. It is recommended that women on acitretin wait a minimum of three years after treatment before attempting to become pregnant, and avoid drinking alcohol while on acitretin and for two months after treatment stops. If isotretinoin were as effective as acitretin, it would be better to give to women of childbearing age. Unfortunately, no studies have directly compared the chemopreventative effectiveness of isotretinoin to acitretin, nor do we completely understand their mechanisms of action. Topical RA (tretinoin/Retin A) used to treat acne vulgaris and skin wrinkling has fewer systemic side effects, but has limited efficacy in clinical studies, and potentially increased cSCC [144,145]. A recent prospective cohort study found that high dietary vitamin A was associated with reduced risk of cSCC [146]. However, 26–28 years of follow-up of the Nurses’ Health and Health Professionals Follow-up studies were needed, as 10–14 years of follow-up was not significant [147]. In addition, levels of vitamin A were several magnitudes greater than the RDA [146]. High total dietary vitamin A, dietary retinol, total retinol, beta-cryptoxanthin, lycopene, and lutein/zeaxanthin were all associated with reduced risk of cSCC, but beta-carotene was not significant. Thus, in humans consuming excess dietary vitamin A is protective, but exogenous oral retinoid treatments are limited to only patients at high risk for cSCC due to detrimental side effects. In addition, resistance to retinoid treatments can occur. Understanding retinoid resistance and the mechanisms by which retinoids act could help produce more targeted treatments.

**Table 3:** Summary of exogenous retinoid effects on cutaneous squamous cell carcinoma (cSCC).

Author (Year)	Animal Model/ Study Population	Tumor Induction	Retinoids	Effect
Verma et al. (1979) [107]	Female CD-1 mice	DMBA/TPA	Topical RA (applied 1 h before TPA treatment)	- Inhibition of ornithine decarboxylase activity - Decreased number of papillomas
Verma et al. (1979) [107]	Female CD-1 mice	DMBA/TPA	Topical RA (applied 24 h before TPA treatment)	- No inhibition of ornithine decarboxylase activity - No decreased number of papillomas
Chen et al. (1995) [112]	Female SENCAR mice	DMBA/MEZ	High dietary RA	- Inhibition of tumor promotion and progression
Chen et al. (1995) [112]	Female SENCAR mice	DMBA/TPA	High dietary RA	Inhibition of tumor progression
Chen et al. (1994) [114]	Female SENCAR mice	DMBA	High dietary RA	- Decreased papilloma formation, but not progression
Passeri et al. (2016) [111]	CRABP-II-knockout C57BL/6 mice	DMBA/TPA		Enhance skin carcinogenesis
Halliday et al. (2000) [119]	Skh:HR-1 (albino)	Solar simulated Ultraviolet radiation	Topical RA	Enhance skin carcinogenesis
Halliday et al. (2000) [119]	Skh:HR-2 (lightly pigmented)	Solar simulated Ultraviolet radiation	Topical RA	Increased skin carcinogenesis
Kligman et al. (1996) [120]	Hairless mice	Solar simulated Ultraviolet radiation (UVB + UVA)	Topical tretinoin	Inhibition of skin carcinogenesis
Kligman et al. (1981) [121]	lightly pigmented variety mice	Ultraviolet light	- Topical RA	No effect on skin carcinogenesis
Kelly et al. (1989) [124]	Skh-hr1	broad-band light (280–700 nm)	- Oral vitamin A - Etretinate	No effect on skin carcinogenesis
Harwood et al. (2005) [138]	Organ transplant patients		Oral acitretin	Prevention of cSCC reoccurence
Kadakia et al. (2012) [139]	Non-transplant patients		Oral acitretin	- Reduction of the number of tumor - No effect on incidence and time of cSCC development
Brewster et al. (2007) [141]	Aggressive cSCC patients		Oral isotretinoin (13-cis RA) with interferon alpha	No effect on cSCC reoccurrence
Weinstock et al. (2012) [144]	cSCC patients		Topical tretinoin	Ineffective on cSCC risk reduction
Weinstock et al. (2009) [145]	cSCC patients		Topical tretinoin	Increased mortality
Fung et al. (2003) [147]	Nurses’ Health and Health Professionals Follow-up studies		High dietary vitamin A (10–14 years follow-up)	No effect on cSCC risk (short follow up period)
Kim et al. (2019) [146]	Nurses’ Health and Health Professionals Follow-up studies		High dietary vitamin A (26–28 years follow-up)	Reduced risk of cSCC

## 4. Altered Vitamin A Metabolism in cSCC

Both UVA and UVB light reduced retinol and retinyl esters levels in the skin of SKH-1 hairless mice, rabbits, and cultured human keratinocytes [103,148,149,150]. However, UVB exposure reduces ddretinyl esters less than retinyl esters in cultured human keratinocytes [103]. In addition, UVB exposure induced ddretinol synthesis in an in vitro assay [103]. Patients with cSCC and AK also have increased ddretinol/retinol ratios in skin [151]. The exact role of ddretinoids in skin following UVA/B exposure is unclear. Tafrova et al. argue that physiological doses of ddretinoids better protect against high dose UVA/B-induced apoptosis [103]. In contrast, Torma et al. argued that ddretinoids have similar effects as retinoids and regulate similar genes [88,152]. They argue that ddretinoids are just a backup system in the skin. Note that ddretinol is increased by etretinate and reduced by isotretinoin [153,154]. Since the etretinate derivative acitretin is better at cSCC prevention than isotretinoin, the ddretinoids may be better at protecting the skin from UV damage and cSCC prevention. However, future studies are needed to better identify the role of ddretinoids in the skin.

Many vitamin A metabolism proteins are altered in cSCC. RBP1 was reduced with increased severity of cSCC [111]. LRAT activity and expression was reduced in cultured human cSCC cell lines [155,156,157], and following acute exposure to UVB [158,159]. This reduced LRAT activity leads to increased retinol, ddretinol, RA, and ddRA [156]. However, *LRAT* mRNA levels increased following chronic UVB, but were not altered in the BCC or cSCC tumors from Ptch1^+/−^/SKH-1 mice [160]. *LRAT* mRNA also increased in trichoblastomas (hair follicle tumor) caused by mouse papillomavirus (MmuPV1) [161]. DHRS9 increased following acute UVB in SKH-1 mice and MmuPV1-induced trichoblastomas [158,159,161]. In contrast, *DHRS9* message levels decreased following chronic UVB exposure in DMBA/TPA and UVB-induced cSCC and in cultured skin cancer stem cells [160,162]. *DHRS3* message levels also decreased following chronic UVB and in cSCC tumors from Ptch1^+/−^/SKH-1 mice [160]. ALDH1A2 moved from the lower epidermis (basal layer) to the upper epidermis (granulosum layer) following acute UVB in SKH-1 mice [158,159]; but mRNA levels in whole tissue homogenates were not altered by chronic UVB or in cSCC tumors from Ptch1^+/−^/SKH-1 mice [160]. *ALDH1A1* and *ALDH1A2* were also greater in regressing KAs than in cSCC [116]. CRABP2 increased following acute UVB in SKH-1 mice, DMBA-induced KAs that were regressing, and MmuPV1-induced trichoblastomas [116,158,159,161]. However, CRABP2 decreased in human cSCC lesions as well as DMBA/TPA and UVB-induced cSCC tumors, and following chronic UVB exposure in mouse models [111,160]. In contrast, Collins and Watt [73] found high immunoreactivity of CRABP2 in DMBA/TPA induced papillomas and cSCC, but they did not quantify their results. The expression of CYP26A1 increased in the middle of the epidermis (spinosum layer) following acute UVB in SKH-1 mice [158,159]; human sun exposed skin; and the precursor lesion actinic keratosis (AK) [163]. *CYP26B1* was also increased as DMBA-induced KAs were regressing [116]. In contrast, CYP26A1 decreased in human malignant cSCC lesions [163]; and *CYP26A1* and *CYP26B1* mRNA levels decreased after chronic UVB exposure and in cSCC tumors from Ptch1^+/−^/SKH-1 mice [160]. RARA increased within the upper epidermis (granulosum layer) while RARB decreased following acute UVB in SKH-1 mice [158,159]. *RARA* and *RARB* were also greater in regressing KAs than in cSCC [116]. In contrast, *RARB* and *RARG* were reduced following chronic UVB, but not in cSCC tumors from Ptch1^+/−^/SKH-1 mice [160]. *RARA*, *RARB1*′, and *RARG* message levels were also lower in human cSCC lesions than BCC lesions [164]. Combined, these studies suggest that the expression of many RA synthesis and degradation enzymes, binding proteins, and receptors are altered by cSCC, with increased levels seen in early disease and reduced levels seen as the disease progresses. Reduced DHRS9 and CRABP2 in cSCC suggest reduced synthesis of RA in cSCC. Additional evidence for reduced RA in cSCC is the reduction of RA target genes LRAT, DHRS9, DHRS3, CRABP2, and CYP26A1 [50,64,65,85,86,87,88,89,90,91]. This reduced RA could lead to less differentiation, more proliferation, and ultimately more severe cSCC, as was seen in the *Crabp2^tm1Ipc^* null and *Cyp26a1* overexpressing mice [111,115]. Understanding how retinoid metabolism is altered in cSCC allows one to target therapies to increase endogenous RA and/or ddRA synthesis. This may produce fewer side effects if RA/ddRA is made in the specific cell it is needed.

## 5. Retinoid Resistance

Retinoid resistance is common in cancer, and understanding the specific mechanisms involved in each cancer will result in better treatments [165,166,167]. Resistance occurs by several mechanisms, which boil down to less RA available in the cell or altered RA signaling. Less RA occurs by reduced retinol uptake, reduced RA synthesis, excess RA catabolism, or increased retinol efflux. As discussed in the last section, DHRS9 and CRABP2 decreased in cSCC [111,160,162]. The drop in *DHRS9* message levels seen in DMBA/TPA induced cSCC was due to an increase in the long noncoding RNA AK144841, which inhibits *Dhrs9* [168]. In addition, UV exposure reduced retinol and retinyl esters levels, [148,149], but it is unknown if this is due to altered influx or efflux in the cell or direct damage to these light sensitive molecules. Altered retinoid signaling can occur by reducing the CRABP2:FABP5 ratio to direct RA to PPARD/B, reducing RARs, or altering the expression of coactivators [166,169]. Collins and Watt [73] saw high levels of CRABP2 and FABP5 in DMBA/TPA-induced cSCC. However, others found reduced CRABP2; and *Crabp2^tm1Ipc^* null mice developed more severe cSCC [111,160]. COLO16 human cSCC cultured cells are resistant to RA and expressed little CRABP2 [170]. Raising the CRABP2:FABP5 ratio by increasing CRABP2 and/or reducing FABP5 did not make these cells sensitive to RA. Retinoid resistance also occurred in *Crabp2^tm1Ipc^* null mice [111]. Additional studies are needed to better determine changes in the CRABP2:FABP5 ratio in cSCC. Retinoid resistance is caused by the hypermethylation of DNA in the promoter of RARs and other genes, resulting in gene silencing in many cancers [171]. In a mouse model of oral SCC, Tang et al. [172] found that combining a methyltransferase inhibitor with low dose RA resulted in reduced tumor number and grade. Methylation induced gene silencing is a physiological mechanism used to regulate hair follicle stem cells as well as terminal differentiation in the epidermis [173]. DNA methyltransferase 1 (DNMT1), DNMT3a, DNMT3b, 5-methyl cytosine, global methylation, and methylation activity were all seen in the epidermis of SKH-1 mice and they were all increased with UVB exposure in a time dependent manner [174]. Increased methylation was also seen in biopsies from human patients with cSCC. These results suggest that increased methylation induced gene silencing occurs during photocarcinogenesis. Increased methylation was seen in the CRABP2 promoter in humans with higher-grade cSCC tumors [111]. Methylation may also explain the reductions in RARB and RARG following chronic UVB, however, we did not find any alterations in the methylation status of RARB 48 h after an acute dose of UVB (Suo and Everts, unpublished observation). The lack of reduced RARs in UVB-induced cSCC suggests that methylation of RARs may not be the major mechanism of retinoid resistance in cSCC. The RAR coactivator tripartite motif protein 16 (TRIM16) was decreased in human AK and cSCC due to increased protein degradation [175]. In contrast, topical treatments with the histone deacetylase inhibitor valproic acid did not impact the effects of topic tazarotene or isotretinoin in UV exposed C3.Cg/TifBomTac hairless mice. Thus, altered coactivators, but not corepressors, may be involved. Overall, these studies suggest that altered retinoid metabolism and signaling in cSCC may limit the use of retinoids to treat this cancer. To date, we see long noncoding RNA, increased DNA methylation, and reduced coactivators contributing to this resistance. However, future studies to identify mechanisms involved may find better treatments than just retinoids alone.

## 6. Prognostic Value of Altered Vitamin A Metabolism

Altered levels of RA metabolism proteins may have prognostic potential in cSCC, however, limited studies have been done specifically in cSCC. Increased LRAT is associated with poor prognosis in melanoma [176] and colorectal cancer [177]. However, reduced LRAT was seen in invasive bladder cancer [178]. Reduced DHRS9 was associated with reduced capacity to synthesize RA in colon cancer cells [179] and poor prognosis in patients with colorectal and oral cancers [180,181]. Thus, the reduction of DHRS9 in cSCC may also predict outcomes. ALDH activity is high in cancer stem cells (CSC; aka tumor initiating cell) and is used to isolate these cells from a number of solid organ tumors [182,183,184,185,186]. Human tumors with increased ALDH1A1 positive cells measured at the protein level are associated with increased risk of recurrence in non-small cell lung cancer [187], enhanced invasiveness in nasopharyngeal carcinoma [188], and poor survival in bladder cancer [189], papillary thyroid carcinoma [190], head and neck cancer [191], esophageal SCC [192], colorectal cancer [193], and breast cancer [194]. On the other hand, high ALDH1A1 levels predicted better outcomes in gastric cancer [195]. Reduced ALDH1A2 predicts poor outcomes in prostate cancer [196] and oropharyngeal SCC [197]. Oropharyngeal SCC with high levels of both ALDH1A2 and CRABP2 predicted better outcomes [197]. High ALDH1A3 leads to poor outcomes in gliomas [198], glioblastoma [199], gallbladder [200], and gastric cancers [195]. However, high *ALDH1A3* mRNA predicted both greater survival and improved reaction to B-RAF/MEK inhibitor treatment in B-RAF-mutant metastatic melanoma [201]. In addition to increasing RA levels, ALDH activity protects CSC by reducing reactive oxygen species and metabolizing chemotherapy drugs [185,186]. Increased RARG was associated with poor prognosis in colorectal cancer [202] and hepatocellular carcinoma [203]. Overall, these studies suggest that LRAT, DHRS9, ALDH1A1, ALDH1A2, ALDH1A3, and RARG levels may be useful biomarkers of numerous advanced cancers that are more likely to recur and/or metastasize, leading to poor survival. In general, higher levels of LRAT, ALDH1A1, ALDH1A3, and RARG predicted poor outcomes, while lower levels of DHRS9 and ALDH1A2 predicted poor outcomes. High LRAT leads to greater storage of retinyl esters and less RA synthesis [156]. Lower DHRS9 and ALDH1A2 also suggest lower RA. However, ALDH1A1 and ALDH1A3 have multiple roles and their increased expression does not mean there is increased RA in these tumors. It is not known which retinoid metabolism protein may be predictive of poor outcomes in cSCC. Finding markers that can predict outcomes allows for more targeted therapy. Plus, retinoid responsiveness may be better predicted by knowing the expression levels of CRABPs and RARs.

## 7. Summary and Conclusions

In summary, the research suggests that retinoids do prevent cSCC. However, this requires decades of excess consumption of vitamin A from animal and plant sources. Intake of vitamin A supplements and beta-carotene were not beneficial. Pharmacological levels of synthetic retinoids had mixed effects, with low dose acitretin being the most beneficial to organ transplant patients. Retinoid metabolism and signaling is altered in cSCC, which may explain some of these mixed effects of exogenous retinoids. Retinoid treatments inhibit EGF and WNT signaling, but other mechanisms may also be involved. 

## 8. Gaps and Future Directions

While much is known about the interactions between retinoids and cSCC, there is still more to learn. We still do not know all of the mechanisms by which retinoids act, especially in the areas of UVB-induced cSCC and immunosuppression. The physiological role of ddretinoids in vivo is also still not clear. It is also not clear how UVB and cSCC development alter retinol and retinyl esters levels as well as their metabolism to RA. Long noncoding RNA and DNA methylation reduced DHRS9 and CRABP2, respectively, but it is not known if restoring these proteins will reverse cSCC. Restoring CRABP2 was not beneficial in one cell model of cSCC [170], but this should be tested in vivo. Future studies could combine retinoid treatments with methylation inhibitors, as was successful in other cancers [172]. Park et al. identified a compound that specifically bound CRABP1 and inhibited mutant RAS signaling [93]. Should we focus on developing specific ligands for CRABP1 and/or CRABP2 in place of RARs? If we know how other proteins in retinoid metabolism are regulated, we could similarly target them to increase endogenous RA and/or ddRA synthesis. This may produce fewer side effects than exogenous retinoids as RA/ddRA would be produced locally where needed. Wu et al. found a compound that increased the expression of some retinoid metabolism genes [160].

Finally, future studies are needed to identify altered retinoid metabolism proteins that have prognostic value in determining patients who might benefit from synthetic retinoid or other treatments. For example, if a patient had a RAS mutation and expressed CRABP1 or CRABP2, then retinoid treatments may be beneficial to specifically reduce this overactive EGF signaling. However, treatment may not be effective if the CRABPs are low, or RAS is not overactive. 

## Figures and Tables

**Table 1:** Natural retinoids [21,22,23,25,26,27,28,29,30,31,32,33,34,35,36,37].

Retinoid	Function	Binding Protein	Synthesizing Enzyme	Transcription Factor	Catabolizing Enzyme	Maximum Absorption
Retinyl esters	Diet and storage		LRAT and DGAT1			
Retinol	Circulation	RBP1-4				325 nm
Retinal	Active in vision	RBP1 and 2	SDRs			383 nm
All-trans-RA	Active in transcription for most functions	CRABP 1, CRABP 2, and FABP5	ALDH1A1, ALDH1A2, ALDH1A3	RARA, B, G	CYP26A1, B1, and C1	350 nm
ddretinyl esters	Storage form		CYP27C1, LRAT			
ddretinol		RBP1 and RBP4	CYP27C1			350 nm
ddretinal	Active in vision, shifts light wavelength		CYP27C1, RDH1/16, RDH10			401 nm
dd-RA	Active in transcription for most functions	CRABP2	CYP27C1	RARA, B, G, RXRA		370 nm

**Table 2:** Clinical uses of retinoids [4,38,39].

Retinoid	Brand Name	Category: Form	Major Use
Retinol, Retinal, Retinyl esters			Cosmetic
Tretinoin (atRA)	Retin A^TM^	1st gen: topical	Acne vulgaris, fine wrinkling, mottled hyperpigmentation, and tactile roughness skin
Isotretinoin (13cRA)	Accutane^TM^, Isotrex	1st gen: oral	Nodulocystic acne and recalcitrant acne
Acitretin (Etretinate)	Neotigason^TM^, Soriatane^TM^	2nd gen: oral	Severe plaque and pustular psoriasis
Tazarotene	Zorac^TM^, Tazorac^TM^	3rd gen: topical	Acne vulgaris and psoriasis (less than 20% body surface area)
Adapalene	Differin^TM^	3rd gen: topical	Acne vulgaris
Bexarotene	Targretin^TM^	3rd gen: oral and topical	Cutaneous T-cell lymphoma
Talarozole	Rambazole^TM^	Cyp26 inhibitor	Ichthyosis
Alitretinoin		3rd gen: oral and topical	Topical: AIDS-associated actinic keratosis
			Oral: chronic eczema in Europe

## Data Availability

Not applicable.
